# P182: Detection of legionella pneumophila DNA from environment of hospitals

**DOI:** 10.1186/2047-2994-2-S1-P182

**Published:** 2013-06-20

**Authors:** O Chubukova, O Kovalishena, A Blagonravova

**Affiliations:** 1 Epidemiology, Nizhniy Novgorod State Medical Academy, Nizhniy Novgorod, Russian Federation

## Introduction

The risk of infection and epidemiological surveillance and control of legionellosis depends on the challenges of detecting the Legionella pneumophila DNA in the hospital environment.

## Objectives

The aim was to compare the various hospital departments in according to the features of existence of L. pneumophila in the environment of hospitals.

## Methods

We conducted real-time PCR of 963 swabs from 40 objects of the environment, 156 samples of water from the centralized water supply and swimming pools in 27 departments of 10 healthcare setting in the Nizhny Novgorod Region.

## Results

We have distinguished the following types of the departments.

Type 1: territories of high risk of health-care associated legionellosis due to high susceptibility of patients, usage of artificial lung ventilation and moisturing the oxygen and air mixture. It includes intensive care units, surgical, obstetrical departments, operating theaters. Type 1 is characterized by the high contamination of environment by L. pneumophila DNA (7,9±1,3 per 100 tests), by presence of reservoirs – the oxygen moisturizers and water taps (12,0% and 11,4% positive results relatively), by a frequent isolation of Legionella pneumophila from water (7,1±1,3 per 100).

Type 2: departments with patients suffering from outpatient pneumonias: therapeutic, pulmonological, thoracic departments. They are characterized by the low contamination of environment (2,2±1,0 per 100) and a frequent isolation of L. pneumophila from water (4,7±1,5 per 100).

Type 3: departments of high risk of nosocomial legionellosis. They have favorable conditions for L. pneumophila and air-borne mechanism of transmission by means of aerosol-producing facilities. They are balneological departments which have a high contamination of environment (14,7±1,4 per 100); reservoirs of L. pneumophila such as the Jacuzzi, therapeutic baths and showers (25,9% and 22,4% positive results relatively); a rare isolation of L. pneumophila from water (3,8±1,4 per 100 tests).

## Conclusion

That is, there are different types of hospital departments for the risk of legionellosis depending on the frequency of detection of Legionella DNA the hospital environment, data about patients, their treatment and diagnostics. Different departments require different approaches to organizing and conducting surveillance of legionellosis.

## Disclosure of interest

None declared

